# Supplementary material for: Structure and Inhibition of the Human Na+/H+ Exchanger SLC9B2
Source: Int J Mol Sci. 2025 Apr 29;26(9):4221. doi: 10.3390/ijms26094221 (PMC12072577; doi:10.3390/ijms26094221)
Supplement: Supplementary file 1 [file ijms-26-04221-s001.zip › ijms-3578197-supplementary.pdf]

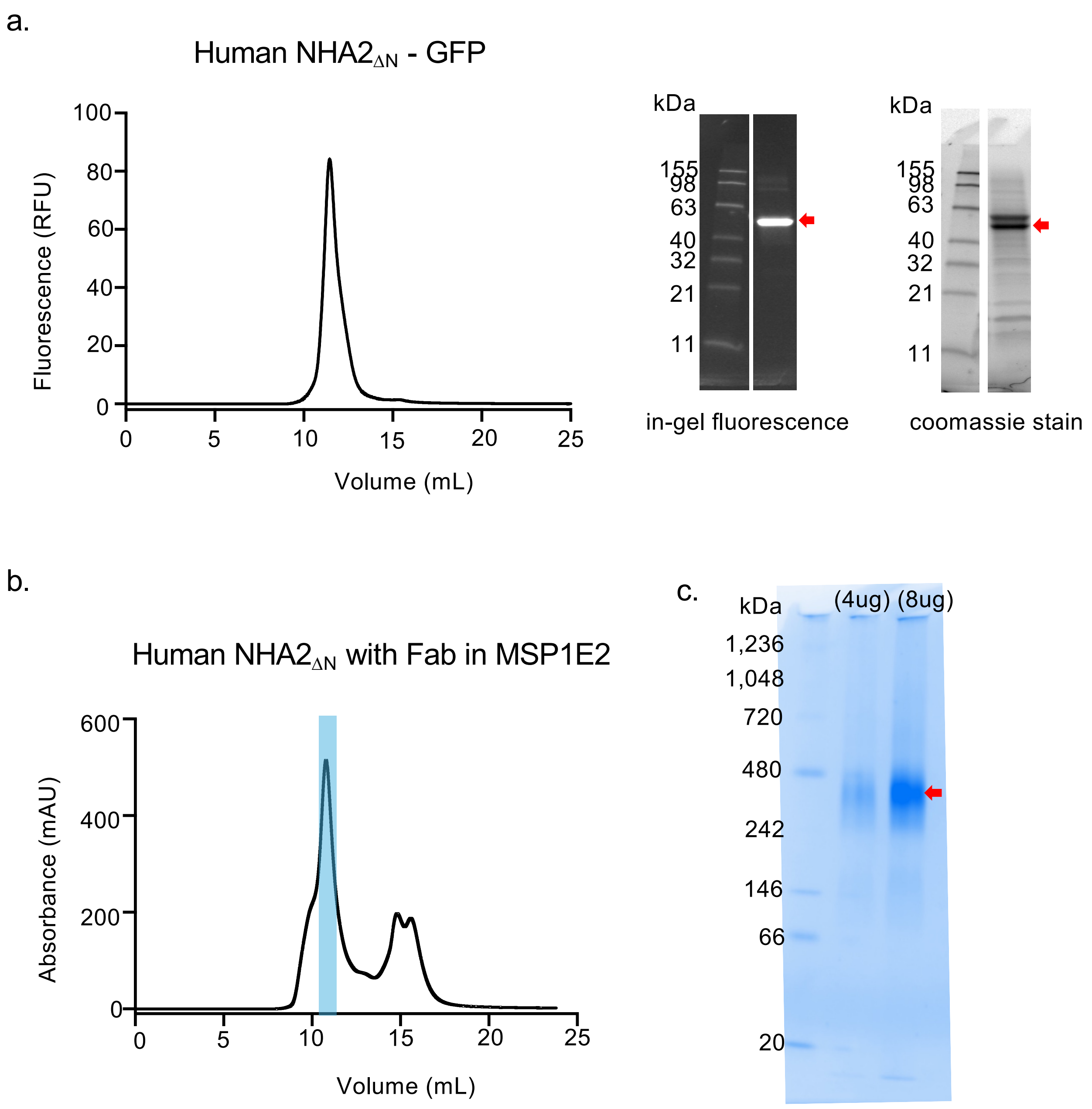

Figure S1. (**a**) Purified human NHA2$_{\Delta N}$– GFP. Size-exclusion chromatography (SEC) profile and SDS-PAGE gel showing the in-gel fluorescence (left) and Coomassie-stained (right) of a human NHA2$_{\Delta N}$–GFP (inset). The arrow indicates the peak corresponding to the purified human NHA2$_{\Delta N}$– GFP. (**b**) SEC profile of human NHA2$_{\Delta N}$–Fab complex reconstituted into MSP1E2 nanodiscs, with the blue-shaded area highlighting the elution volume used for structural experiments. (**c**) Blue native-PAGE analysis of reconstituted NHA2$_{\Delta N}$–Fab complex into MSP1E2. Coomassie staind 4-16% gradient blue-native gel. The arrow indicates the human NHA2$_{\Delta N}$–Fab complex.

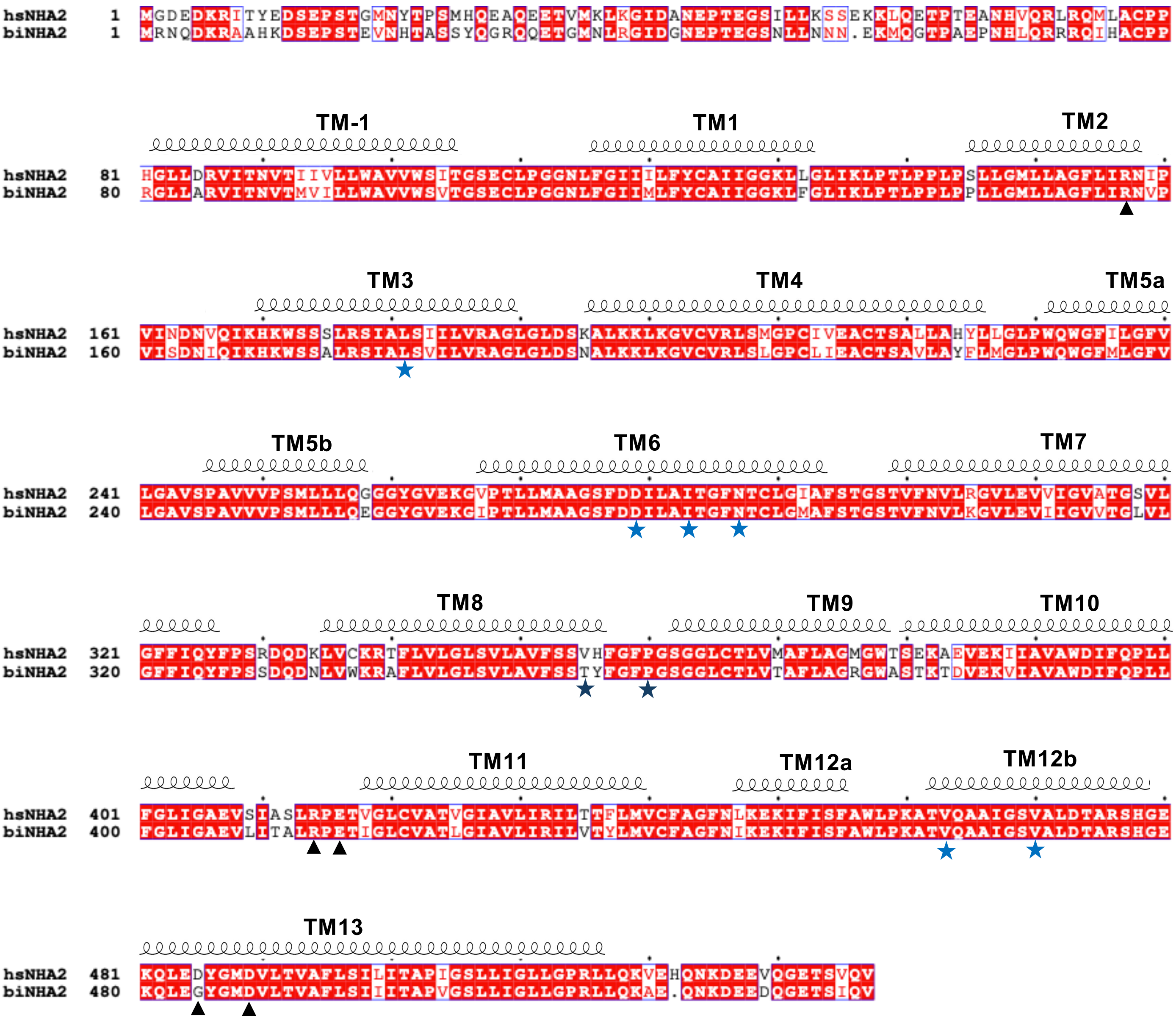

Figure S2. Sequence alignment of human NHA2 and bison NHA2. The following are the accession ID's for human NHA2 (Q86UD5), bison NHA2 (A0A6P3HVI0). Residues with over 90% sequence identity are indicated by red background. Residues interacting with fab are indicated with triangles (black) and Phloretin with star (blue).

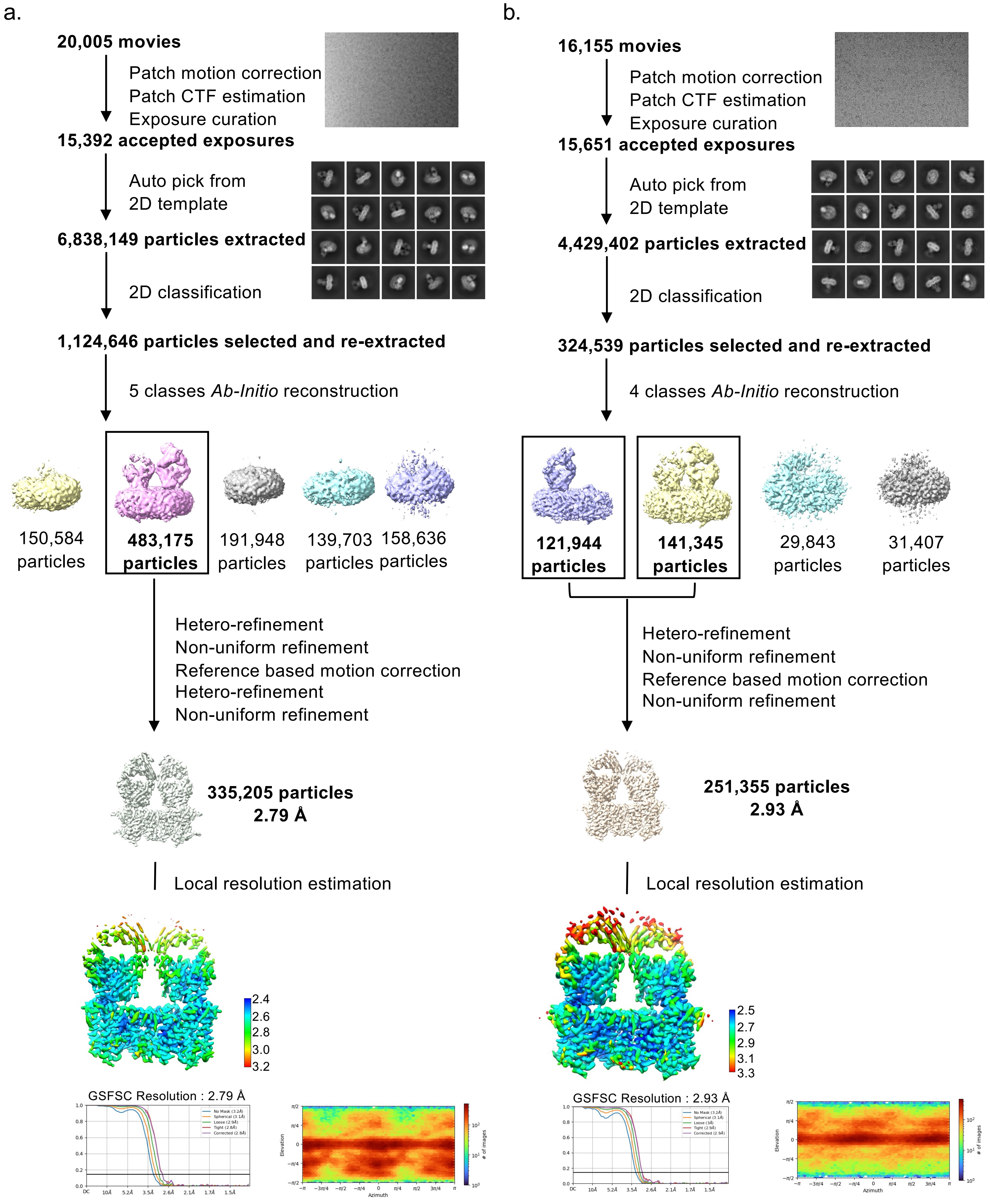

Figure S3. Cryo-EM processing workflow of human NHA2ΔN-Fab complex. (**a**) The dataset of NHA2-Fab in nanodisc contained 20,005 movies that were corrected by MotionCor2 [54] and CTFFind [55]. After reference-based picking with Topaz 6,838,149 particles were picked. Several rounds of 2D classifications yielded in good 2D classes of 1,124,646 particles. 3D classification and 3D refinement of model resulted in an electron density map of 2.8 Å. The electron density map was subjected to non-uniform refinement local refinement, per particle CTF and Bayesian polishing. A final resolution of 2.79 Å was achieved at gold-standard FSC (0.143). (**b**) As in a., for NHA2$_{\Delta N}$-Fab incubated with phloretin prior to freezing on cryo-EM grids. The Fab was masked out during local refinement. The final reconstruction was obtained from 251,355 particles and had an overall resolution of 2.93 Å based on gold standard FSC at 0.143.

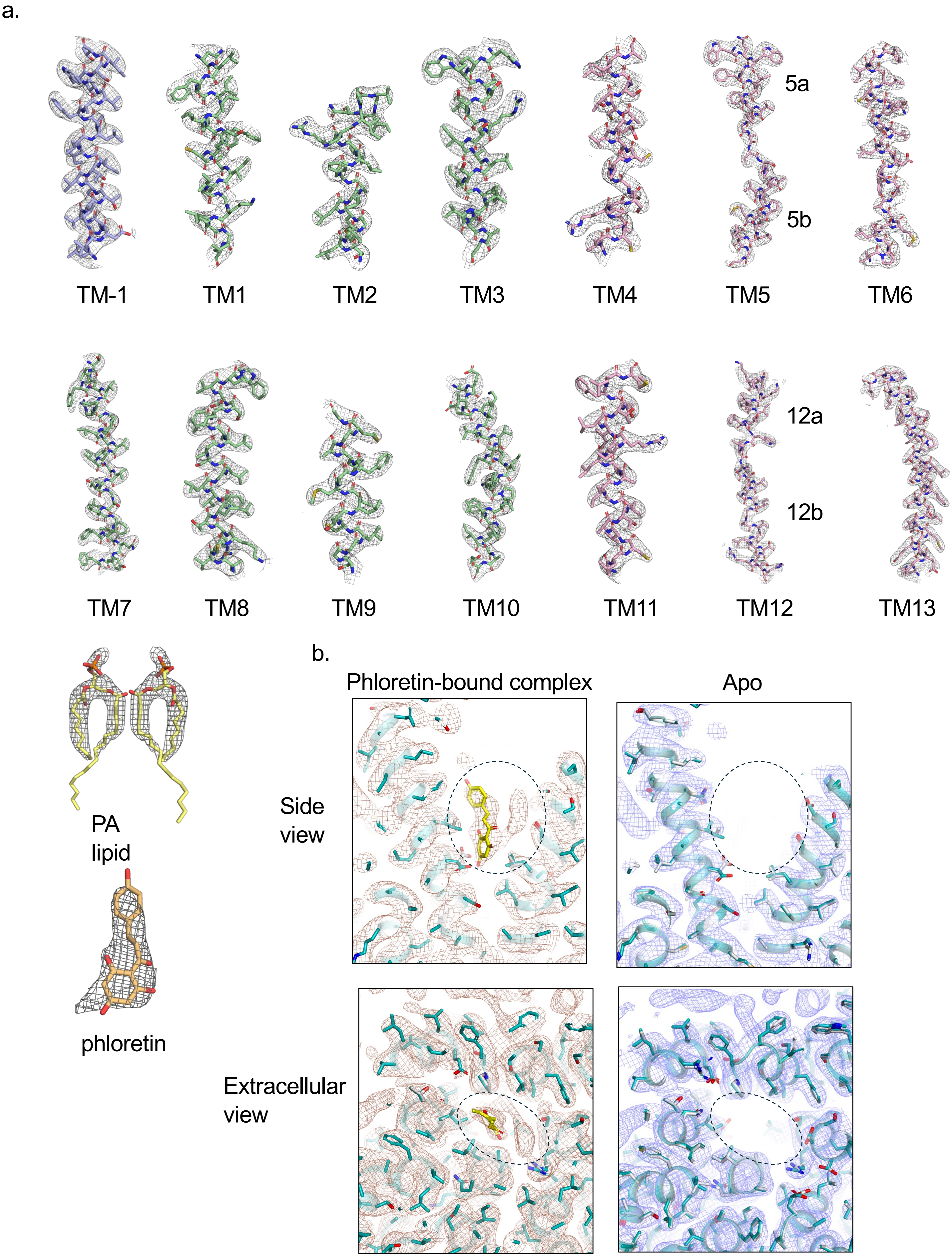

Figure S4. Cryo-EM density of human NHA2. (**a**) Cryo-EM density map and model are shown for all the transmembrane segments for human NHA2 in dimer domain (pale green), transport domain (light pink), and TM-1 helix (light purple), PA lipid (yellow), Phloretin (orange), all protein shown as sticks. (**b**) Map of phloretin bound NHA2 (left) and apo NHA2 (right) at the same threshold showing the same high-quality of density for the side chains, no signal for the phloretin in the apo NHA2 cryo-EM map (right).

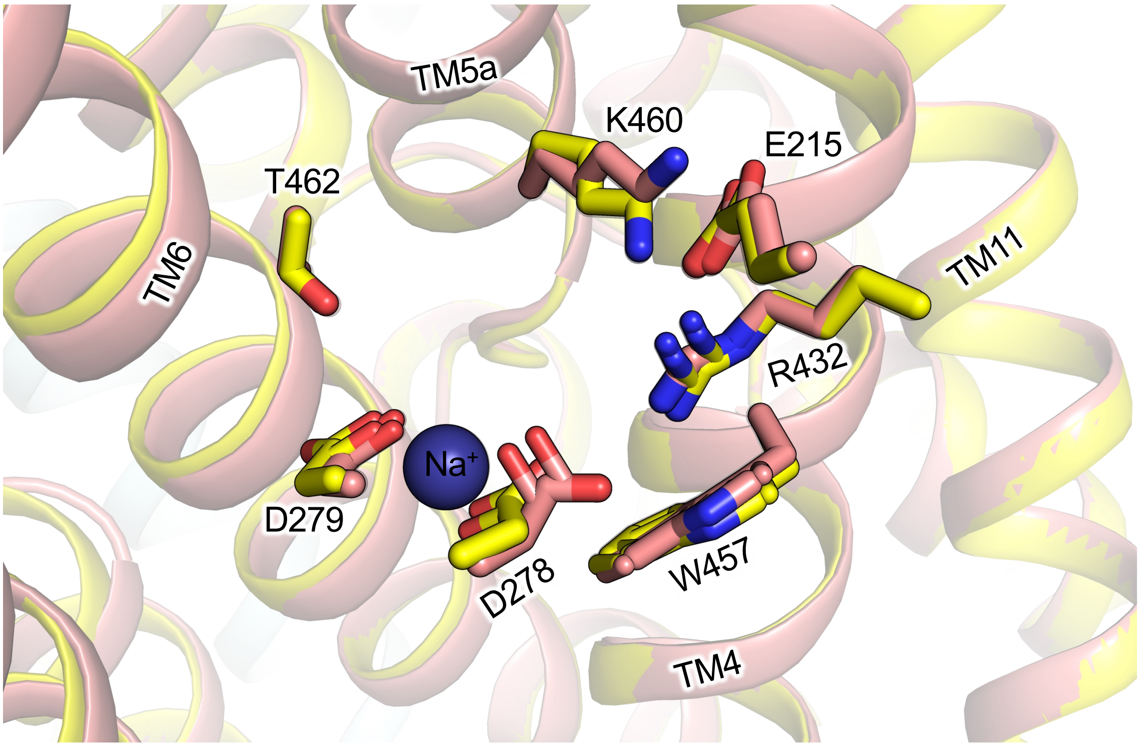

Figure S5. The ion binding site predicted by AF3. Predicted human NHA2 apo (pink) and human NHA2 Na^+ bound structure (yellow).

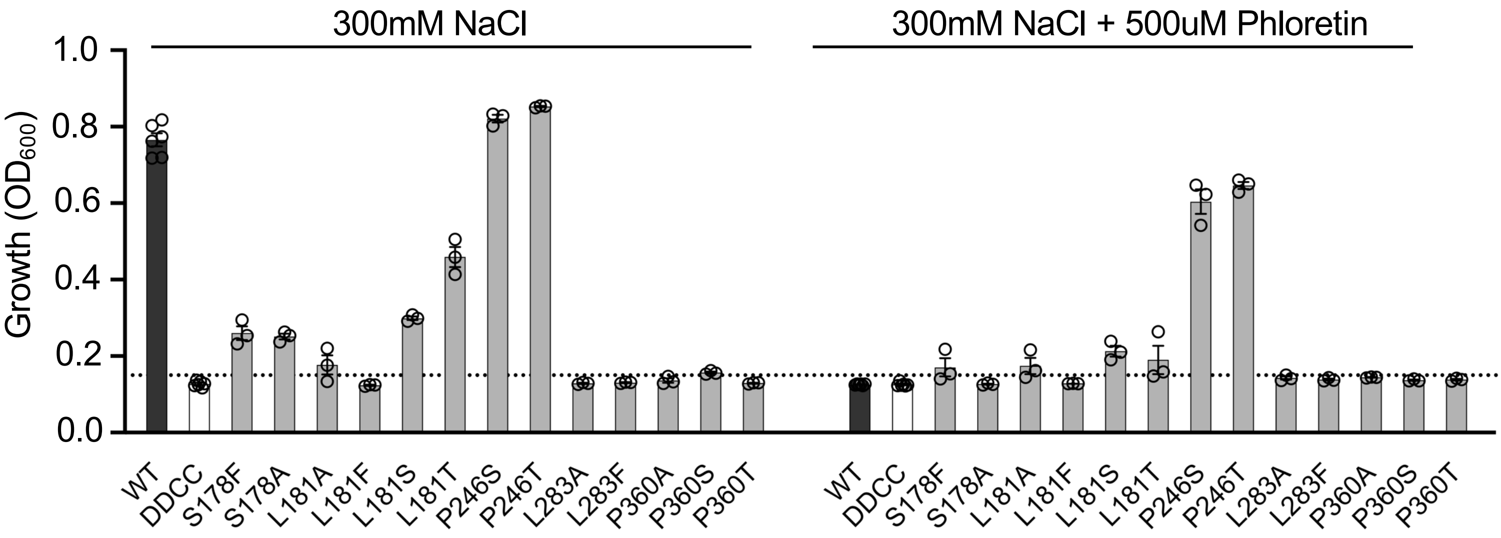

Figure S6. Salt sensitivity of *S. cerevisiae* AB11c cells expressing either human NHA2 wild type (WT) or mutants thereof—double cysteine DDCC (D278C and D279C), S178A/F, L181/A/F/S/T, P246/A/S/T, and P260A/S/T. Cells were grown in –URA medium supplemented with 300 mM NaCl as indicated. Growth was monitored for 4 days at 30 °C.

	human NHA2$_{\Delta N}$-Fab complex apo<br>EMD-53377 , PDB 9QUB	Phloretin-bound complex of human NHA2$_{\Delta N}$ with Fab<br>EMD-53384, PDB 9QUW
**Data collection and processing**		
Magnification	130	130
Voltage (kV)	300	300
Electron exposure (e–/Å^2)	62.4	65.7
Defocus range (μm)	2.0 to 0.6	0.4 to 1.8
Pixel size (Å)	0.648	0.65
Symmetry imposed	C2	C2
Initial particle images (no.)	6,638,149	4,429,402
Final particle images (no.)	332,205	251,355
Map resolution (Å)	2.79	2.93
FSC threshold	0.143	0.143
Map resolution range (Å)	2.4-3.2	2.5-3-3
**Refinement**		
Initial model used (PDB code)	Human NHA2$_{\Delta N}$-Fab with Phloretin	Bison NHA2$_{\Delta N}$ (7p1k),<br>AF3 model for Fab
Model resolution (Å)	2.7	2.9
FSC threshold	0.143	0.143
Map sharpening B factor (Å^2)		
Model composition		
Non-hydrogen atoms	12,879	12,966
Protein residues	1,685	1,666
Ligands	2 LPP	2 LPP<br>2 G50
B factors (Å^2)		
Protein	140.82	129.60
Ligand	131.72	118.19
R.m.s. deviations		
Bond lengths (Å)	0.004	0.008
Bond angles (°)	0.605	0.671
Validation		
MolProbity score	2.23	2.32
Clashscore	22.45	24.47
Poor rotamers (%)	0.21	0.07
Ramachandran plot		
Favored (%)	94.29	93.06
Allowed (%)	5.71	6.94
Disallowed (%)	0.00	0.00

Table S1. Cryo-EM data collection, refinement and validation statistics of human NHA2$_{\Delta N}$-Fab complex apo and phloretin-bound complex of human NHA2 with Fab.
